# Does Mouthwash Use Affect Oral Cancer Risk? A Comprehensive Systematic Review and Meta-Analysis

**DOI:** 10.7759/cureus.77123

**Published:** 2025-01-08

**Authors:** Ashwini M Madawana, Mohamad Arif Awang Nawi, Liszen Tang, Akram Hassan, Mohd Fadhli Khamis

**Affiliations:** 1 School of Dental Sciences, Hospital Pakar Universiti Sains Malaysia, Kota Bharu, MYS

**Keywords:** meta-analysis, mouthwash, oral cancer, prisma, risk-factors, systematic review

## Abstract

Studies indicate a strong correlation between the length and degree of alcohol and tobacco use and the risk of oral cancer (OC). However, there has been debate concerning the usage of mouthwashes and associated higher risk of OC for many years. The purpose of this study was to gain insight into how using mouthwash influenced the risk of OC. The Preferred Reporting Items for Systematic Reviews and Meta-Analyses (PRISMA) protocol was used when searching the PubMed/MEDLINE, Scopus, and Web of Science databases. Observational studies that addressed the relationship between mouthwash use and OC and involved adult or older adult populations were included. The Newcastle-Ottawa Scale was employed to check the methodological quality, and random effects meta-analysis, along with other subgroup analyses and meta-regression, were utilized to synthesize quantitative data. Out of 5,132 papers identified, 15 case-control studies comprising 6,515 cases and 17,037 controls were included in the review. Seventeen effect measures from these 15 studies were included in the meta-analysis. For individuals who used mouthwash three or more times a day, the pooled OR for OC was 1.00 (95% CI: 0.79-1.26; n = 17 studies). Among those who had used mouthwash for more than 40 years, the OR was 1.30 (95% CI: 1.58-4.82; p = 0.05; n = 2 studies). Some studies suggest that frequent mouthwash use may increase the risk of OC. Given the biological plausibility of this link, we exercise caution in interpreting these findings. It is important to note the limited research on the frequency and duration of mouthwash use. Thus, for the strengthening of the evidence for a possible dose-response effect of mouthwashes on OC risk, we suggest that future research should be focused on the frequency, duration, and substance of mouthwashes in depth.

## Introduction and background

The scientific community continues to be highly divided over the existence or absence of a link between the use of mouthwashes and the development of oral squamous cell carcinoma (OSCC) [1]. The oral cavity is the environment of many indigenous bacteria and is connected to health problems in humans [2]. Maintaining oral hygiene is challenging due to the absence of time and stress. Various ways can be used to minimize ailments related to oral hygiene, such as caries and periodontal problems. Good oral health has become an essential part of our daily life nowadays. Several awareness programs carried out by oral health care providers have made people know that poor oral hygiene can be one of the roots of many health problems [3].

The first step to good oral hygiene is tooth brushing to get rid of plaque, which is a colorless layer made by the mass of microorganisms on the tooth surface [4]. To achieve better oral hygiene, apart from tooth brushing, mouthwash and other interdental cleaners can be utilized for the effective cleaning of teeth [5]. Therefore, these days, people have an incomplete feeling without using mouthwash even after brushing their teeth thoroughly [6]. The bacteria that reside in dental plaque, such as lactobacilli and mutans streptococci, generate acids. These acids are deposits of microorganisms [7]. These objects produce waste with a pH of 4, which is lower than the pH of the oral cavity (5.5). This indicates that the wastes are highly acidic and may lead to the demineralization of teeth [8]. Additionally, mouthwashes help maintain the oral health regimen but should be used with caution due to their acidic nature. Mouthwashes are used as antiseptic treatments to lower the oral cavity’s bacteria load. Moreover, it is a therapy tool for other inflammatory illnesses such as gingivitis, periodontitis, and oropharyngeal diseases [9].

The mouthwashes that do not have alcohol and have fluoride are also the ones that help to stop tooth decay. There are many different therapeutically active ingredients in the mouthwash; alcohol is one of them, and the concentration of alcohol ranges from 0% to 27%. In the current paper, it is reported that alcohol-based mouthwashes can result in oral carcinomas [10]. It is generally accepted that alcohol consumption raises the risk for several cancers, including OSCC. The most common form of alcohol found in mouthwashes, ethanol, metabolizes in the mouth, producing the carcinogen acetaldehyde. Accarbamyl acetaldehyde can damage DNA, interfere with the DNA repair process, and promote uncontrolled growth of cells, all of which lead to the occurrence of cancer [3]. Additionally, alcohol increases the oral mucosa’s permeability to other carcinogens, such as tobacco smoke and environmental contaminants that enter the body. Since both nicotine and alcohol interact to affect the oral epithelium, it has been traditionally recognized that a history of both increases the risk of OSCC [6,9]. Oral cancer (OC) is a disease that does not terminate and has the potential to develop over a long period [11]. Nevertheless, the cause of the phenomenon can also be other factors like drinking, smoking, or betel nut chewing and using alcohol-based mouthwashes, which are yet to be confirmed. A couple of studies have proved the correlation between alcohol-based mouthwashes and OSCC [12].

This led to a potentially debatable association between OSCC and the long-term, consistent use of mouthwashes with alcohol content. Although alcohol-containing mouthwashes have been the primary focus of studies, the potential carcinogenic effects of mouthwashes, in general, warrant investigation due to other chemical components such as chlorhexidine and hydrogen peroxide, which may also contribute to oral mucosal irritation and cytotoxicity. Furthermore, analyzing any mouthwash use, irrespective of alcohol content, allows for a comprehensive understanding of the potential risk factors for OC beyond alcohol alone. There have only been a few contradicting epidemiological studies conducted. Thus, this study intends to identify any significant relationships between the various schools of thought held by the numerous reviewers and the use of mouthwashes and OCs.

## Review

Methodology

Literature Search

The Preferred Reporting Items for Systematic Reviews and Meta-Analyses (PRISMA) protocol is followed in the present study [13]. On May 8, 2024, online databases was searched for the publications, using the following search strategy: PubMed: (("mouthwash"[MeSH Terms] OR "mouthwash" [All Fields] OR "mouth rinses"[MeSH Terms] OR "mouth rinses" [All Fields]) AND ("oral neoplasms"[MeSH Terms] OR "oral neoplasms" [All Fields] OR "oral cancer" [All Fields] OR "oral squamous cell carcinoma"[MeSH Terms] OR "oral squamous cell carcinoma" [All Fields] OR "mouth neoplasms"[MeSH Terms] OR "mouth neoplasms" [All Fields])) AND ("systematic review"[Publication Type] OR "meta-analysis as topic"[MeSH Terms] OR "meta-analysis"[Publication Type] OR "meta-analysis"[All Fields]); Web of Science: TS=("mouthwash" OR "mouth rinse" OR "mouth rinses") AND TS=("oral cancer" OR "oral neoplasms" OR "oral squamous cell carcinoma" OR "mouth neoplasms") AND TS=("systematic review" OR "meta-analysis"); Scopus: TITLE-ABS-KEY(mouthwash OR "mouth rinse" OR "mouth rinses") AND TITLE-ABS-KEY("oral cancer" OR "oral neoplasms" OR "oral squamous cell carcinoma" OR "mouth neoplasms") AND TITLE-ABS-KEY("systematic review" OR "meta-analysis"). This search resulted in a total of 5,132 articles.

Studies Selection

The inclusion criteria for this study were studies examining the association between mouthwash use and the risk of OC; both mouthwashes containing alcohol and no alcohol were included; studies published in peer-reviewed journals; studies with human participants of any age group; studies reporting outcomes related to the incidence, prevalence, or risk of OC associated with mouthwash use; and studies published in English. The exclusion criteria for this study were animal studies, laboratory studies, reviews without original data, letters, editorials, commentaries, and conference abstracts; studies that focus solely on other oral health outcomes not related to OC; studies that assess the efficacy of mouthwash in treatment rather than its association with OC risk; and studies without a follow-up duration.

In this study, the PICO framework is applied to identify studies that investigate the association between mouthwash use (exposure/intervention) and the risk of OC (outcome) in human populations (population). Studies comparing different levels or types of mouthwash use to nonusers (comparison) and reporting relevant cancer-related outcomes were considered.

Operational Definition of OC

OC is a term used to refer to a variety of tumors that originate in the oral cavity. It is also known as mouth cancer or cancer of the oral cavity. While the cheeks, the gums, the roof inside the mouth, the tonsils, and the salivary glands might potentially be the initial site, the lips, tongue, and floor of the mouth are where they most frequently appear.

Article Screening and Data Extraction

**Figure 1:**
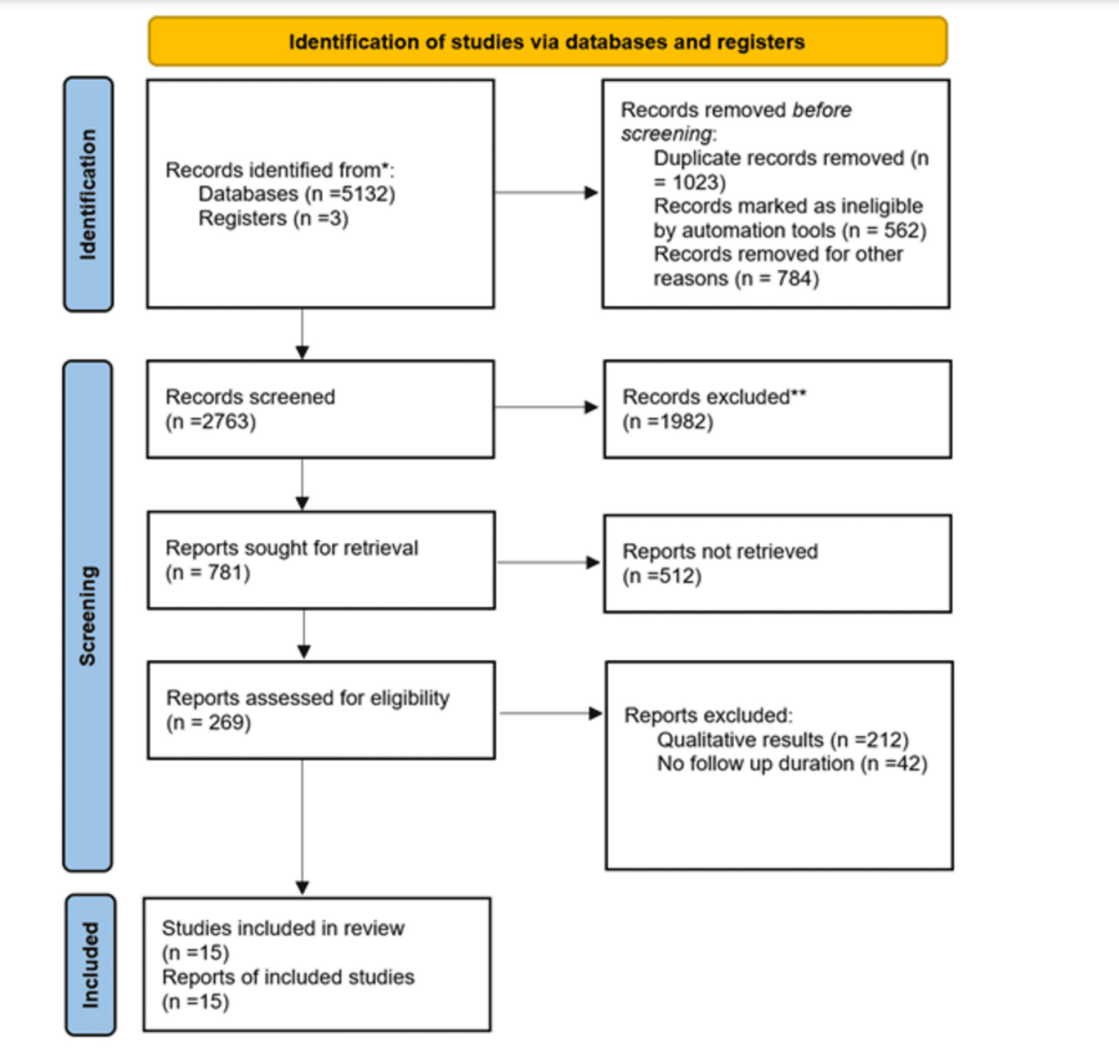
PRISMA flowchart design PRISMA, Preferred Reporting Items for Systematic Reviews and Meta-Analyses

Article screening: The database searches yielded a cumulative total of 5,132 records. A total of 1,023 duplicate records, 562 records flagged as ineligible by automation technologies, and 784 records eliminated for other reasons were excluded before screening. After eliminating duplicate entries, a total of 2,763 records were analyzed. A total of 1,982 records, out of the initial 2,763, were excluded from the analysis. The reasons for their exclusion were as follows: 932 records involved animal studies, 85 were case series, 34 were related to diagnosis, 433 relied on guidelines, 76 were solely focused on congenital heart disease, 90 were related to other diseases, 119 were preclinical studies, 134 were reviews, and 79 were unrelated treatment records. Out of the initial 781 reports, 512 could not be retrieved. Publications containing qualitative data were excluded from the remaining 269 publications (n = 212), and studies with no follow-up duration were excluded (n = 42). This resulted in the final report of 15 studies that are reported in this review as seen in Figure 1.

Data extraction: Two independent reviewers, AMM and MAAN, conducted the screening of titles and abstracts, independently applying the inclusion and exclusion criteria. Any disagreements were resolved through discussion, and when consensus could not be reached, a third reviewer, LT, was consulted for arbitration. For data extraction, AMM and MAAN independently extracted data using a standardized form, with the extracted data cross-checked for accuracy.

The full-text articles were evaluated using the Ottawa scale criteria, employing a standardized form for data extraction [14]. Publications were assessed as a methodological quality indicator and ranked on a scale of low, medium, or high. This ranking was determined by considering various factors such as reporting bias, performance, and selection. The preference for selection was assessed based on the descriptions of the inclusion and randomization criteria. The assessment of performance bias was conducted by examining the descriptions of a control arm and the concealment of allocation. Distinct scores were allocated to selective reporting, incomplete data management, industry sponsorship, and biased reporting. The topics of reporting uniformity and eligibility restrictions were deliberated in several teleconferences. Prior to reaching a conclusion, a tertiary author examined disparities in the evaluators' ratings.

Meta-Analysis

For the meta-analysis, Stata 14.0 (StataCorp LLC, College Station, Texas, USA) was utilized. The DerSimonian-Laird random effects technique was selected due to the substantial (77.1%) heterogeneity assessed by the I² test. In order to determine a likely dose response, subgroup analyses were performed on studies that provided information on mouthwash use frequency and duration. Additionally, the subgroup was assessed based on the variables taken into account during the confounding correction and the kind of control (hospital or community).

Results

Characteristics of Included Studies

Table 1 shows the summary of the studies investigating the association between mouthwash use and OC risk. All studies featured the same design: controversies when it comes to the relation between the causes of the newly wicked (melphalan) and the accidental death (that patient died out of chemotherapy, not cancer). Two studies were classified as multicentric [15,16]: the ARCAGE studies, which were conducted in several European nations [17], and a collection of case-control studies that were both published and unpublished in North America, the European Union, and Asia. Other studies that also relied on these well-known multicentric data were not included so that there would be no duplication of samples from the same survey [15,16,18-22]. Researches were performed in the USA [22-27], Australia [27], Pakistan [28], Brazil [29], Italy [30], China [31], and India [32]. Most studies have relied on hospital controls, and only three out of seven have used community controls [16,32]. The oropharynx was the topic of the eight studies, while six studies included the oral cavity, and in a single study, the larynx was also indicated [26,33].

**Table 1:** Summary of studies investigating the association between mouthwash use and OC risk NOS, Newcastle-Ottawa Scale; OC, oral cancer; OSCC, oral squamous cell carcinoma

Study	Country	Recruitment period	Study setting	Study design	Sample size of the study	Range of age/median	Exposure categories	Outcome of the study	Confounding factors are taken into consideration during sample restriction and matching	Assessment of risk rias (NOS)	Conclusions
Ahrens et al. (2014) [15]	Multicentric	2002-2005	Hospital	Case-control	1983 cases (945 mouth/oropharynx cancer cases); 1981 controls	59.8	Mouthwash use frequency	Oropharynx, hypopharynx, pharynx, larynx, or esophagus	Gender, age, study location, smoking status, length of time spent drinking alcohol, and cumulative alcohol intake	Minimal risk	Mouthwash use poses minimal risk.
Boffetta et al. (2016) [16]	Multicentric	1982-2013	Hospital and community	Case-control	8,991 cases (2,800) OC cases); 10,090 controls (10,020 OC controls)	15-80	Mouthwash usage; years spent using it: 0 (nonusers), 1-15, 16-35, or 36+ years; use frequency per day: 0 (nonusers), 1-2 times/day	Head and neck cancer	Study center, age, gender, average amount of alcohol consumed, cumulative tobacco smoking (pack-years), and educational attainment	Minimal risk	Mouthwash use poses minimal risk.
Blot et al. (1983) [23]	USA	1976-1978	Hospital	Case-control	206 cases; 352 controls	67	Years (0-4, 5-10, 10-24, ≥25) and length of mouthwash retention, frequency of use, and concentration	Female patients suffering from pharyngeal and OC	Age, race, region, the respondent - whether proximate or not - and smoking habits	Minimal risk	The study suggested a potential link between prolonged use of alcohol-containing mouthwash and an increased risk of OC.
Young et al. (1986) [25]	USA	Unreported	Community	Case-control	237 male and 78 female cases; 240 male controls and 56 female controls	63.2/61.5	Mouthwash use	OC and cancers of the oropharynx and hypopharynx	Smoking practices	Moderate hazard	The study found no significant association between mouthwash use (both containing and not containing alcohol) and the risk of developing OC.
D'Souza et al. (2007) [26]	USA	2000-2006	Community	Case-control	100 cases; 100 controls	≤55, ≥70	Mouthwash usage (regularly)	Oropharyngeal OSCC	Age and sex	Moderate risk	Mouthwash use poses moderate risk.
Alnuaimi et al. (2015) [27]	Australia	2011-2013	Community	Case-control	53 cases; 104 controls	23-89	Regular alcohol mouthwash user	OSCC	Gender, age, and dental prosthesis use	Moderate risk	Mouthwash use poses moderate risk.
Marques et al. (2008) [29]	Brazil	1999-2003	Hospital	Case-control	468 controls (406 mouth cancer controls); 309 cases (168 mouth cancer cases)	40-80+	Use of mouthwash (never, a few times a day, several times a day)	Pharynx and mouth cancers	Gender, age, education level, smoking, alcohol intake, and any other factors about oral health and hygiene	Minimal risk	Mouthwash use poses minimal risk.
Talamini et al. (2000) [30]	Italy	1996-2000	Hospital	Case-control	132 cases; 148 controls	27-86	Mouthwash usage (regularly)	Oral cavity and oropharyngeal cancer	Age, gender, consumption of fruits and vegetables, and smoking	Minimal risk	Mouthwash use, particularly those with low alcohol content, is not associated with an increased risk of OC.
Chang et al. (2013) [31]	China	2010-2013	Hospital	Case-control	316 cases (211 OC cases); 296 controls	20-82	Using mouthwash (with or without alcohol) or not using mouthwash	Oral cavity, oropharynx, hypopharynx, and larynx contain oral OSCC	Gender, age, education level, frequency of alcohol consumption, and pack-year groups for chewing betel quid and smoking cigarettes	Minimal risk	Mouthwash use poses minimal risk.
Winn et al. (1991) [32]	USA	1983-1985	Community	Case-control	572 male and 292 female cases; 832 male and 438 female controls	18-80	Mouthwash use (individuals who, for at least six months, used mouthwash once a week)	Primary occurrence of pharyngeal or OC	Age, ethnicity, level of education, study location, drinking, smoking, and amount of fruit consumed	Minimal risk	Regular mouthwash (with increasing alcohol content) users had an elevated risk of developing OC - 40% higher in men and 60% higher in women compared to nonusers.
Sharma et al. (2020) [34]	India	Unreported	Unreported	Case-control	200 cases; 200 controls	53.4/51.7	Mouthwash use daily	Oral OSCC	Not corrected for confounding bias	Moderate risk	Mouthwash use poses moderate risk.
Mashberg et al. (1985) [35]	USA	1981-1982	Hospital	Case-control	93 cases; 913 controls	40-70+	Mouthwash use (containing alcohol): those who said they used the product at least four times a week were considered to be regular users	Pharyngeal and oral carcinoma	Smoking practices	Moderate hazard	Combined use of alcohol and tobacco had a multiplicative effect, substantially elevating the risk of oral and pharyngeal cancer.
Eliot et al. (2013) [36]	USA	2006-2010	Community	Case-control	512 cases (142 oral cavity cases); 567 controls	56/60.5	Mouthwash use frequency (never; sometimes; at least once a day)	Incident cases of head and neck OSCC	Factors such as age, gender, race, education, smoking, and alcohol intake	Minimal risk	Mouthwash use is associated with head and neck OSCC; however, no difference was observed between the effects of alcohol-containing and nonalcoholic mouthwashes.
Assunção Júnior et al. (2015) [37]	Brazil	Unreported	Hospital	Case-control	34 cases; 22 controls	26-87	Mouthwash usage, mouthwash type, and frequency of use each week	OSCC found in the oropharynx and oral cavity	Age and gender	Moderate risk	Mouthwash use poses moderate risk.
Saira et al. (2019) [38]	Pakistan	2014-2016	Community	Case-control	276 cases; 276 controls	55/52.8	Mouthwash use	Carcinoma in the oral cavity, larynx, hypopharynx, and oropharynx	Age, race, and language	Moderate risk	Mouthwash use poses moderate risk.

Only Sharma et al. [34] Mashberg et al. [35], and Young et al. [25] were matched to the others, but only minimally by gender and age. Besides the confounding factors like tobacco and alcohol consumption, which were taken into consideration in multivariable analysis, other less often fruit and vegetable consumption, ethnicity, and socioeconomic conditions were also part of the confounding variables. Human papillomavirus (HPV) was not incorporated in the regression analyses of the findings of the identified studies. A study narrowed the sample to people who are 40 years old or older and do not smoke. Another investigation was superficial, i.e., it did not match the individuals, it did not have a restriction, and it did not do a multivariable analysis [34].

Risk Bias and Quality Assessment

Risk of bias assessment in the studies included in the systematic review on the use of mouthwash and OC using the Newcastle-Ottawa Scale (NOS) for case-control studies, as shown in Table 2. Eight studies showed a low risk of bias, while seven showed a moderate risk, according to NOS. Since the examined studies present hospital controls, a portion of their methodological flaw is the way they chose their controls. Furthermore, no study disclosed the blinding of controls and patients concerning exposure, which may have led to measurement bias.

**Table 2:** Risk of bias assessment using the NOS for case-control studies Rating scale: 7-9 stars = low risk of bias; 4-6 stars = moderate risk of bias; 0-3 stars = high risk of bias Selection: maximum 4 stars; Comparability: maximum 2 stars; Exposure: maximum 3 stars NOS, Newcastle-Ottawa Scale

Study	Selection				Comparability	Exposure		
	1	2	3	4	1	1	2	3
Ahrens et al. (2014) [15]	★	★			★★	★	★	★
Boffetta et al. (2016) [16]	★	★	★		★★	★	★	★
Blot et al. (1983) [23]	★	★			★★	★	★	★
Young et al. (1986) [25]	★	★				★	★	★
D'Souza et al. (2007) [26]	★	★			★	★	★	★
Alnuaimi et al. (2015) [27]	★	★		★	★	★	★	
Marques et al. (2008) [29]	★	★			★★	★	★	★
Talamini et al. (2000) [30]	★	★			★★	★	★	★
Chang et al. (2013) [31]	★	★		★	★★	★	★	★
Winn et al. (1991) [32]	★	★	★		★★	★	★	★
Sharma et al. (2020) [34]	★	★				★	★	
Mashberg et al. (1985) [35]	★	★			★	★	★	★
Eliot et al. (2013) [36]	★	★	★		★★	★	★	★
Assunção Júnior et al. (2015) [37]	★	★				★	★	★
Saira et al. (2019) [38]	★	★			★	★	★	

Meta-Analysis of Included Studies

**Figure 2:**
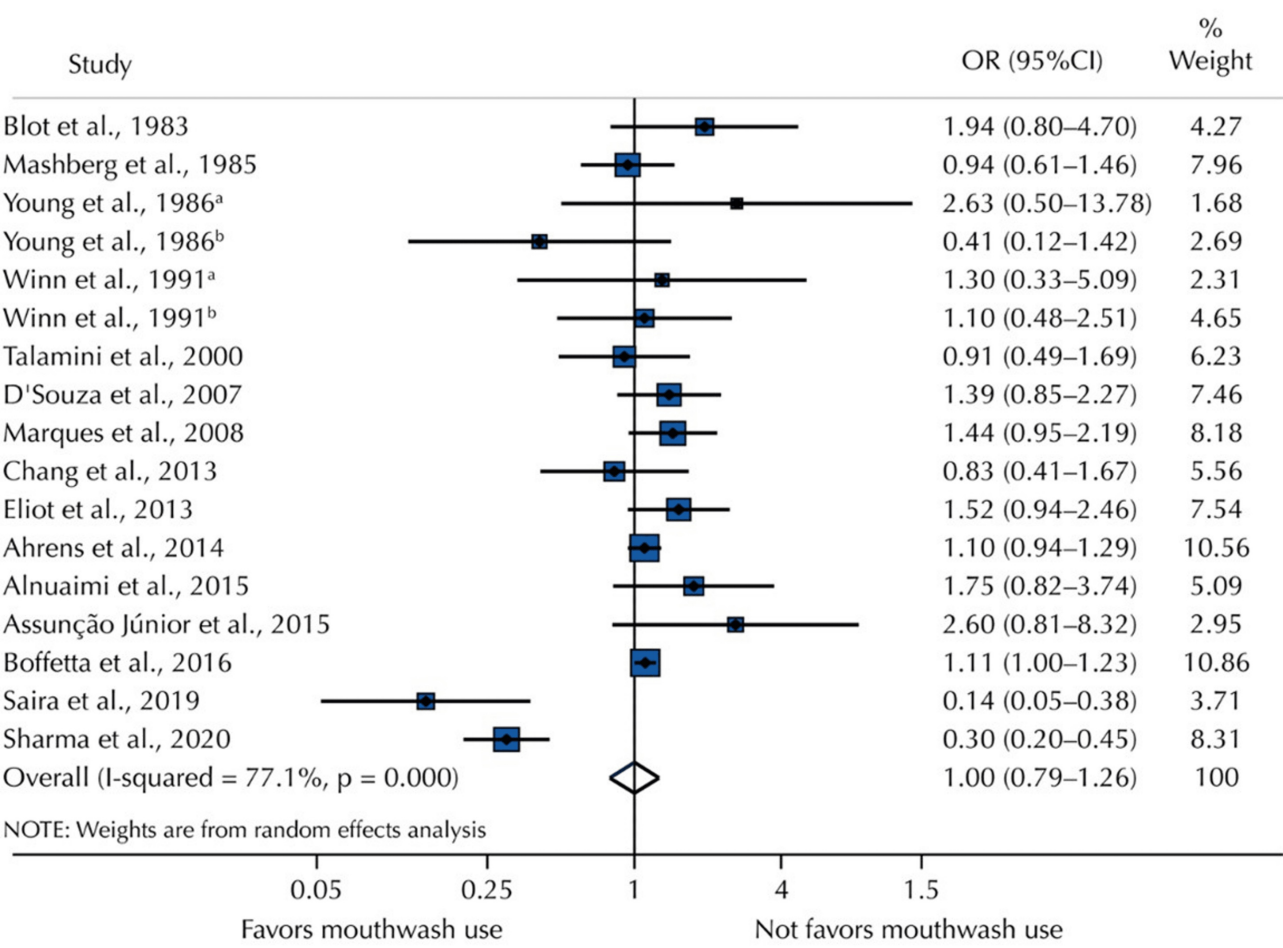
Random effects of mouthwash users versus nonusers on the OR of OC in a meta-analysis Ahrens et al. (2014) [15], Boffetta et al. (2016) [16], Blot et al. (1983) [23], Young et al. (1986) [25], D'Souza et al. (2007) [26], Alnuaimi et al. (2015) [27], Marques et al. (2008) [29], Talamini et al. (2000) [30], Chang et al. (2013) [31], Winn et al. (1991) [32], Sharma et al. (2020) [34], Mashberg et al. (1985) [35], Eliot et al. (2013) [36], Assunção Júnior et al. (2015) [37], Saira et al. (2019) [38] OC, oral cancer

The summary of the 17 ORs from the 15 trials that made up the meta-analysis is displayed in Figure 2. There was significant heterogeneity among trials (I²: 77.1%), and mouthwash use was not linked to overconsumption (OR = 1.00; 95% CI: 0.79-1.26), independent of alcohol level or frequency/duration of use.

**Figure 3:**
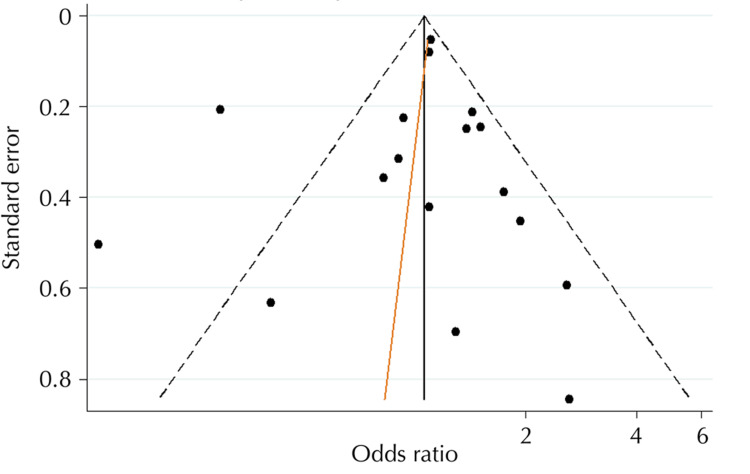
Funnel plot with pseudo 95% confidence limits Ahrens et al. (2014) [15], Boffetta et al. (2016) [16], Blot et al. (1983) [23], Young et al. (1986) [25], D'Souza et al. (2007) [26], Alnuaimi et al. (2015) [27], Marques et al. (2008) [29], Talamini et al. (2000) [30], Chang et al. (2013) [31], Winn et al. (1991) [32], Sharma et al. (2020) [34], Mashberg et al. (1985) [35], Eliot et al. (2013) [36], Assunção Júnior et al. (2015) [37], Saira et al. (2019) [38]

Although the funnel plot (Figure 3) indicates a low likelihood of publication bias due to symmetry in the distribution of studies, the Egger tests were not considered statistically significant (p = 0.651), suggesting that the smaller studies may have had an impact. The modified line for the Egger asymmetry regression test is represented by the orange line.

**Figure 4:**
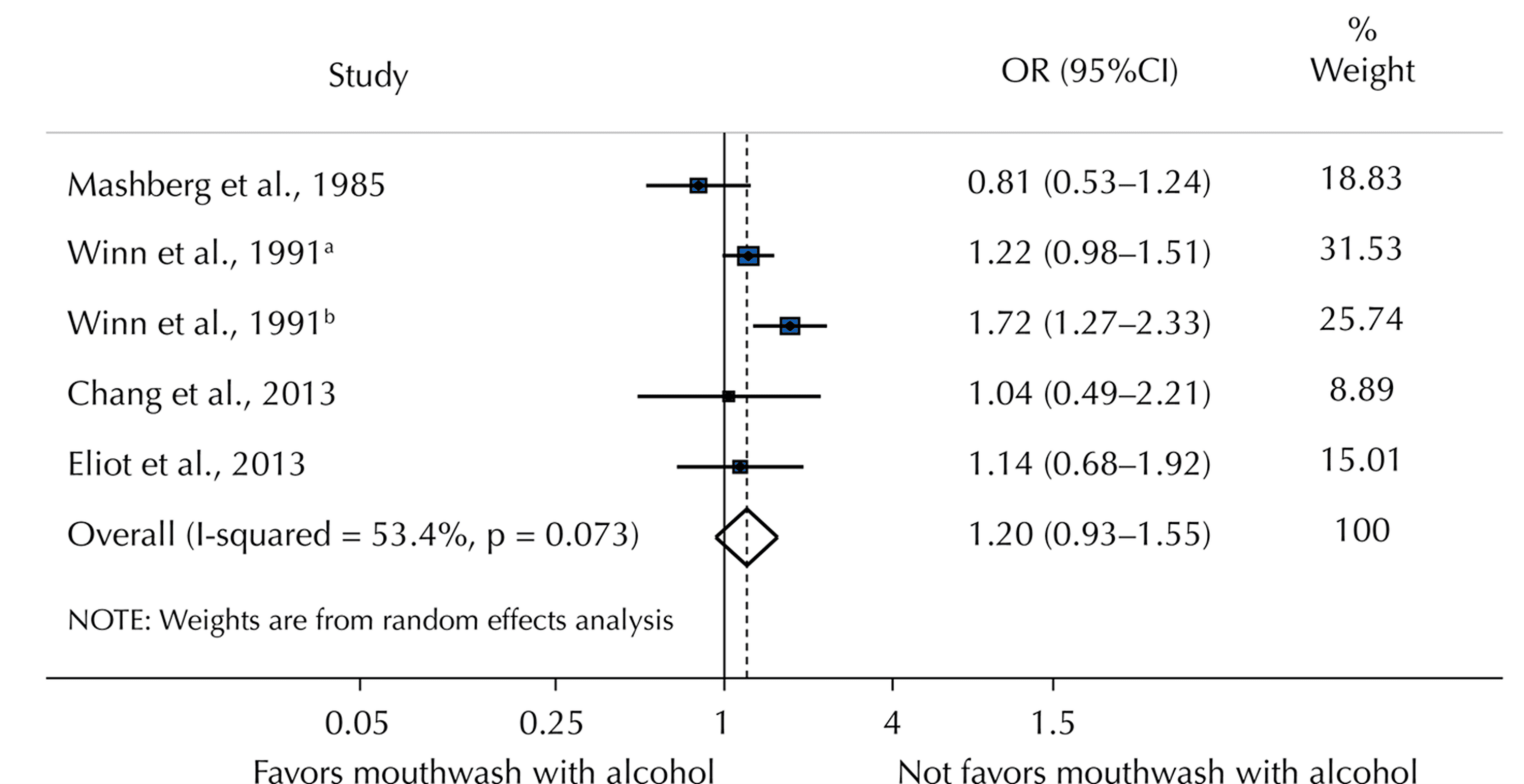
Random effects of the OC OR that favors and does not favor mouthwashes with alcohol Chang et al. (2013) [31], Winn et al. (1991) [32], Mashberg et al. (1985) [35], Eliot et al. (2013) [36] OC, oral cancer

The total weighted random effect grew but remained insignificant when only the five estimated effects (OR) of the study investigations evaluating alcohol-containing mouthwash versus no mouthwash use were taken into account (OR = 1.20; 95% CI: 0.93-1.55) (Figure 4).

Mouthwash use (OR = 1.00; 95% CI: 0.69-1.26) and control type (OR = 1.22; 95% CI: 0.95-1.40) were not observed to be associated with overconsumption based on the subset of covariates taken into account in the confounding adjustment. Regarding mouthwash users’ frequency of consumption, the overall weighted random effect showed statistical significance (OR = 1.35; 95% CI: 1.10-1.54). This demonstrated that using mouthwash for more than 40 years was linked to a 44% higher incidence of OC compared to those who did not (OR = 1.44; 95% CI: 1.10-1.90). Mouthwash usage was not linked to OC when compared to not using it at all (OR = 0.85; 95% CI: 0.65-1.12) and once or twice a day (OR = 1.13; 95% CI: 0.93-1.37). On the other hand, using mouthwash three or more times a day was linked to a higher risk of OC (OR = 2.58; 95% CI: 1.38-4.82).

Mouthwash

Using a total of 17,037 controls and data from 15 case-control studies with 17 OR estimates, this systematic review and meta-analysis found no significant correlation between mouthwash use (any versus none) and the development of OC (OR = 1.00; 95% CI: 0.79-1.26).

Previous Meta-Analyses

Three earlier meta-analyses yielded no conclusive results. Based on the analysis of the 17 studies conducted by Hostiuc et al. [6] on upper aerodigestive tract cancers and their relation to mouthwash use, the researchers concluded that the difference in the risk between the cases and the controls was not significant. Moreover, Aceves Argemí et al. were unable to find a connection between mouthwash and OC in five case-control studies involving mouthwash that contained alcohol or four studies that did not [39]. Similarly, a very implausible nonsignificant relative risk was compiled from nine studies by Gandini et al. Additionally, these writers considered how often and how long people used mouthwash [40]. Recent studies published in the last five years, which have not been included in previous studies, provide new relevant data. These studies have compared to the above research in terms of methodological design and sample size, which could increase the relevance of data. The new data make it possible to compare the contemporary trends in mouthwash composition, including alcohol-free mouthwash that has displaced the alcoholic versions for various reasons.

Frequency and Duration of Mouthwash

Among individuals who used mouthwash three or more times a day, the probability of developing OC was significantly elevated. Specifically, the data revealed that long-term users (those with over 40 years of use) had a 158% higher incidence of OC compared to nonusers (OR = 2.58; 95% CI: 1.1-3.8). Wynder et al. [41] assessed the relative risk based on daily usage frequency (one, two, or three times per day) and found no statistically significant increase in risk with higher daily use frequencies. Similarly, Hostiuc et al. [6] discovered that there was a nonstatistically significant probability difference between the frequency of usage and the development of upper aerodigestive tract cancers. No other meta-analyses that looked at the relationship between dose and response with OC could be found during the investigation [6].

Risk Factors for OC

The factors that are considered to be the risks for OC are tobacco, alcohol, and betel consumption; diet; nutrition; environmental and genetic factors. By conducting subgroup analyses according to the studies that reported both crude and adjusted associations, the likelihood of confounding that influenced the pooled estimates was minimized [42,43]. However, it is impossible to rule out the possibility of unmeasured confounding because it is possible that significant confounding variables, such as socioeconomic conditions, diet and nutrition, alcohol and cigarette consumption, HPV infection (which was not taken into account in any of the studies), and socioeconomic conditions, may have been overlooked (only taken into account in some of the association estimates). Furthermore, residual confounding is likely to be applied if a confounding factor is not adequately characterized or assessed. However, given age increases are linked to an increased risk of OSSC, we can presume that the participants’ mouthwash use time may have been influenced by their age. However, age and other variables that might account for the observed connection have been taken into account in all the studies included in this subgroup analysis [6].

Alcohol Content in Mouthwash

Over the years, they developed this idea because they believed that mouthwashes and overconsumption were related to the alcohol content of these products [44]. The tumorigenesis process that is triggered by human epithelial keratinocytes, which were evaluated in vitro with two commercial mouthwash brands, would cause the cell-killing impact. Two variants of each brand - 95 mg/mL and 214 03 mg/mL, respectively - containing and lacking alcohol have been evaluated on human oral keratinocytes with and without moderate dysplasia. The scientists came to the conclusion that alcohol-based mouthwashes caused broad alterations in the expression of genes in vitro, which were genotoxic to both normal and dysplastic oral keratinocytes [45].

Similar results were also observed in the clinical trials evaluating the effects on the exfoliated oral cells of mouthwashes containing and without alcohol [46]. This suggests that the group that used the alcohol rinse included the cells with the greatest quantity of micronuclei and cellular abnormalities. Because of the surface and internal characteristics of the oral mucosa epithelium, it is difficult to distinguish between damage to DNA and cellular death in desquamated cells of the epithelial layer. Therefore, in order for the genotoxic material to cause damage to DNA and for micronuclei to form during cell division, it must be able to get through the basal layer’s permeability barrier. The number of stem cell divisions in a tissue over the course of a person’s lifetime and the risk of that tissue developing cancer are statistically correlated [47].

In our meta-analysis, the link between using mouthwashes containing alcohol and not using it was not statistically significant (OR = 1.20; 95% CI: 0.85-2.56). A summary of the alcohol content data from nine investigations was provided by the researchers (93-0.55), who also discovered a nonsignificant connection (OR = 1.48; 95% CI: 0.85-2.56). Because of this, the alcohol concentration and composition of mouthwashes were not properly reported in all trials; nonetheless, it is important to note that these nonalcoholic compounds with antibacterial action may also be cytotoxic. Numerous antiseptics with different active ingredients are used in dentistry and are available for a large variety of applications. These goods are classified as cosmetic items; hence, they do not need ingredient declaration. Therefore, we can presume that other components are also implicated in cell damage or oral microflora imbalances, which, in turn, could suppress the immune regulatory mechanisms, thus promoting the formation and progression of OC [39].

Other Chemical Components

The chemicals found in mouthwashes that are most discussed are triclosan, octenidine, delmopinol, triclosan, cetylpyridinium chloride, hyaluronic acid, and natural substances. Although alcohol has been the primary focus of mouthwash-related cancer studies, it is important to highlight the potential role of other chemical components in contributing to oral health risks, including carcinogenic effects. The effects of these substances on the human gingival fibroblasts are inhibited when cellular metabolic activity is reduced by 50% at the concentration required for this purpose (IC50). Two percent chlorhexidine reduced the number of viable cells and increased the proportion of cells affected by acidic conditions. Other, on the other hand, different in vitro studies confirmed these conclusions. The results of the study indicate that cetylpyridinium chloride, even at low concentrations, has a strong cytotoxic effect on human keratinocytes and mouse fibroblasts [48]. Given the widespread use of mouthwashes and their varying chemical compositions, the potential cytotoxicity and tissue damage induced by ingredients such as cetylpyridinium chloride and chlorhexidine warrant further investigation for their relevance to cancer risk. After assessing the cytotoxicity of Listerine, a product including thymol, eucalyptus, methyl salicylate, and menthol, the scientists concluded that all phenolic components may partially or entirely cause cell damage in vitro.

Immune cells, mitochondria, and possibly the brain are all harmed by triclosan. Triclosan was removed from dentifrices by Colgate-Palmolive and the US FDA in 2017. Over-the-counter antiseptic medications no longer contain triclosan or 23 other active compounds since there is inadequate data about their efficacy and safety [49].

Limitations of the Meta-Analysis

The inability to conduct subgroup analyses based on the various amounts of alcohol concentration in the mouthwash, however, was a drawback of our meta-analysis. Alternatively, given the evidence is based solely on in vitro research, we could investigate if the chemicals in the products have a noteworthy impact on OC in the unlikely event that they are unrelated to the alcohol content. Therefore, it is imperative to do fresh research on the amount of alcohol in mouthwashes and their primary constituents in order to have a better understanding of the effects of these substances on human health [50].

In addition, this meta-analysis was the first to apply the GRADEpro GDT for evaluating the quality of the evidence [51]. The evidence quality was graded as low by the tool. Therefore, the primary reason for this result is the way the included studies were designed. The best kind of epidemiological study design to look at this topic is a case-control study; however, it has more biases than cohort and clinical trials. Although the majority of studies have been categorized by the NOS’s criteria as having low or moderate risk of bias, in this case, the likelihood of some bias being caused by confusing measurement and selection bias is so high that the risk of bias is classified as “serious” by GRADEpro. However, because it is not possible to randomize the research, the nonrandomness of case-control studies may have led to an underestimation of the risk of bias in the included studies using the NOS instrument, which was employed in this meta-analysis. However, NOS is one of the most widely used tools, and both its interobserver reliability and content validity are strong [15,52]. The same tool was employed in a recent meta-analysis on the subject; also, NOS yields the same reliability as the Cochrane-recommended ROBINS-I tool, with the only distinction being application. Furthermore, the complexity of the ROBINS-I tool’s use may be the cause of its limited adoption. Another element that lowers the quality of the evidence is the inconsistent outcomes, since some research has demonstrated that using mouthwash can either be protective against OC or advantageous (risk).

## Conclusions

The results of this systematic review and meta-analysis indicated that there was no correlation between mouthwash use and OC, except for those who used it for more than 40 years or three times a day, which seems to suggest a dose-dependent impact. Although there are not numerous studies that take mouthwash frequency into account, these findings should still be interpreted cautiously. To strengthen the evidence for a potential dose-response effect of this exposure on OC risk, we thus advise that future research thoroughly assess the frequency, duration, and substance of mouthwashes.
